# Supplementary material for: SyNDI: synchronous network data integration framework
Source: BMC Bioinformatics. 2018 Nov 6;19:403. doi: 10.1186/s12859-018-2426-5 (PMC6219086; doi:10.1186/s12859-018-2426-5)
Supplement: Supplementary file 5 — Files of the example presented in Additional file 4. This additional file is a zip package that contains all files used in Additional file 4. Detailed descriptions of each of these files are presented in a README file included in this zip package. (GZ 30411 kb) [file 12859_2018_2426_MOESM5_ESM.gz › SyNDITools/Meme2Fimo/meme_4.9.0_4.tar.gz!/meme_4.9.0/doc/gomo.html]

GOMO - a Gene Ontology association tool for motifs


# The MEME Suite

## Motif-based sequence analysis tools

### Usage:

`gomo [options] <go-term database> <scoring file>+`

### Description

The name `GOMO` stands for "Gene Ontology for Motifs."
The program searches in a set of ranked genes for enriched GO terms
associated with high ranking genes. The genes can be ranked, for example,
by applying a motif scoring algorithms on their upstream sequence.

The p-values for each GO-term are computed empirically by shuffling
the gene identifiers in the ranking (ensuring consistancy across species)
to generate scores from the null hypothesis. Then q-values are derived
from these p-values following the method of Benjamini and Hochberg
(where "q-value" is defined as the minimal false discovery rate at which
a given GO-term is deemed significant).

The program reports all GO terms that receive q-values smaller than a
specified threshold, outputting a gomo score with emprically calculated
p-values and q-values for each.

### Input

#### GO Term Database

a collection of GO terms mapped to to the sequences in the scoring
file. Database are provided by the webservices and are formated using a
simple tsv-format:  
*"GO-term" "Sequence identifiers separated by tabulator"*   
The exception to this rule is the first line which instead contains the
url to lookup the gene ids. The url has ampersands (&) replaced with
&amp; and the place for the gene id marked by the token
"!!GENEID!!" .

#### Scoring File

a XML file which contains for each motif the sequences and their
score. The XML file uses the
CisML
schema. When scoring data is available for multiple related species GOMO
can take multiple scoring files where the true sequence identifiers have
been mapped to their orthologs in the reference species for which the
go-term database was supplied.

### Output

GOMO will create a directory, named `gomo_out` by default.
Any existing output files in the directory will be overwritten. The
directory will contain:

- An XML file named `gomo.xml` providing the results in a
  machine readable format.
- An HTML file named `gomo.html` providing the results in a
  human readable format.

The default output directory can be overridden using the
--o or --oc options
which are described below.

Additionally the user can override the creation of files altogether by
specifying the --text option which outputs to
standard out in a tab seperated values format:  
"Motif Identifier" "GO Term Identifier" "GOMO Score" "p-value"
"q-value"

By default GOMO calculates the ranksum statistics on the
*p*-values of each gene given in the
CisML
input file. Using the option --gs switches
the focus from the *p*-values to the scores. Any sequence failing to
provide a *p*-value will prompt GOMO to abort the calculations. The
same happens when any of the genes in the CisML file lacks a score
attribute and --gs was activated.

### Options:

| Option | Parameter | Description | Default Behaviour |
| --- | --- | --- | --- |
| General Options | | | |
| --- | --- | --- | --- |
| --o | name | Create a folder called name and write output files in it. This option is not compatible with -oc as only one output folder is allowed. | The program behaves as if `-oc memechip_out` had been specified. |
| --oc | name | Create a folder called name but if it already exists allow overwriting the contents. This option is not compatible with -o as only one output folder is allowed. | The program behaves as if `-oc memechip_out` had been specified. |
| --dag | go dag file | Path to the optional Gene Ontology DAG to be used for identifying the most specific terms in the gomo xml output so they can be highlighted in the html output. |  |
| --text |  | Output in tab separated values format to standard output. Will not create an output directory or files. |  |
| --motif | id | Use only the motif identified by id. This option may be repeated. | All motifs are used. |
| --shuffle\_scores | n | Number of times to shuffle the sequence = score assignment and use the shuffled scores to generate empirical p-values. |  |
| --score\_E\_thresh | n | Threshold used on the gene score *E*-values above which all *E*-values become maximal in order to reduce the impact of noise. Subsequently, this results in all genes having *E*-values above the threshold to obtain the same rank in the ranksum statistics. The threshold will be ignored when gene scores are used (--gs). |  |
| --t | n | Threshold used on the q-values above which results are not considered significant and subsequently will not be reported. To show all results use a value of 1.0. | A threshold of 0.05 is used. |
| --min\_gene\_count | n | Filter out GO-terms which are annotated with less genes. | A value of 1 is used which shows all results. |
| --gs |  | Indicates that gene scores contained in the cisml file should be used for the calculations. | Use the gene *p*-values. |
| --nostatus |  | Suppresses the progress information. |  |
| --verbosity | 1\|2\|3\|4\|5 | Set the verbosity of status reports to standard error. | The normal verbosity level of 2 is used. |
